# CpATG8, a Homolog of Yeast Autophagy Protein ATG8, Is Required for Pathogenesis and Hypovirus Accumulation in the Chest Blight Fungus

**DOI:** 10.3389/fcimb.2019.00222

**Published:** 2019-07-10

**Authors:** Liming Shi, Jinzi Wang, Rui Quan, Feng Yang, Jinjie Shang, Baoshan Chen

**Affiliations:** ^1^State Key Laboratory for Conservation and Utilization of Subtropical Agro-bioresources, College of Life Science and Technology, Guangxi University, Nanning, China; ^2^Jiangsu Key Laboratory for Microbes and Functional Genomics, College of Life Sciences, Nanjing Normal University, Nanjing, China

**Keywords:** *cpatg8*, autophagy, hypovirus, virulence, chestnut blight fungus

## Abstract

Autophagy is a degradation system in the cell, involved in the turnover of cellular components, development, differentiation, immune responses, protection against pathogens, and cell death. Autophagy is induced by nutrient starvation, in which cytoplasmic components and organelles are digested *via* vacuoles/lysosomes. In this study, by using electron microscopy, we observed that hypovirus CHV1-EP713 infection of *Cryphonectria parasitica*, the causative agent of chestnut blight disease, caused proliferation of autophagic-like vesicles. This phenomenon could be mimicked by treating the wild-type strain of the fungus EP155 with the autophagy induction drug rapamycin. Some of the hypovirulence-associated traits, including reduced pigmentation and conidiation, were also observed in the rapamycin-treated EP155. Quantitative reverse transcriptase polymerase chain reaction (qRT-PCR) revealed that genes involved in autophagy were up-regulated in expression. Deletion of *cpatg8*, a gene encoding a homolog of ATG8 in *Saccharomyces cerevisiae*, resulted in attenuation of virulence and reduction in sporulation, as well as accumulation of the double-stranded viral RNA. Furthermore, virus-encoded p29 protein was found to co-localize with CpATG8, implying that the viral protein may interfere with the function of CpATG8. Taken together, these findings show that *cpatg8* can be regulated by the hypovirus and is required for virulence and development of the fungus and accumulation of viral dsRNA in chestnut blight fungus.

## Introduction

Autophagy is a conserved cellular process of eukaryotic cells that degrades intracellular protein complexes and organelles in the vacuole or lysosome (Klionsky et al., 2016; Liu et al., 2016; Yin et al., 2016). Autophagy has diverse physiological functions in the regulation of energy and nutrient metabolism, organelle quality control, removing misfolded proteins (Yin et al., 2016), and the development of filamentous fungi (Khan et al., 2012; Voigt and Pöggeler, 2013b; Liu et al., 2016). Among many molecular elements, ATG8, a ubiquitin-like protein, is a key element of autophagy pathway (Klionsky et al., 2016). In *Saccharomyces cerevisiae*, it was reported that ATG8 is conjugated to the lipid phosphatidyl ethanolamine (PE) and required for autophagosome formation (Nakatogawa et al., 2007). ATG8 is localized to preautophagosomal structures (PAS), autophagosomes, and autophagic bodies (Suzuki et al., 2001). In filamentous fungi, autophagy has been shown to be involved in virulence, cellular growth, development, and environmental stress (Pollack et al., 2009; Bartoszewska and Kiel, 2011; Klionsky et al., 2016; Liu et al., 2016). In *Fusarium graminearum, FgATG8* was found to function in the formation of aerial mycelium and formation of reproductive structures, nutritional use of storage lipid droplets, and infection (Josefsen et al., 2012).

Autophagy could be induced by various abiotic and biotic stresses including pathogen infection (Hayward and Dinesh-Kumar, 2011). In addition, the canonical function of autophagy may play a role in antivirus activities (Dagdas et al., 2016; Clavel et al., 2017; Hafrén et al., 2017; Haxim et al., 2017). However, different animal and plant viruses have developed diversified strategies to evade or hijack the autophagy pathway to promote their own infection or transmission (Dong and Levine, 2013; Chen et al., 2017). Although the role of autophagy in host–virus interactions in animals and plants has been studied to some extent, functions of autophagy in the fungus–virus interface remain to be understood.

Chestnut blight caused by *Cryphonectria parasitica* is a well-known forest disease. Infection with hypoviruses, a group of plus sense RNA viruses, attenuates virulence of this fungus (Dawe and Nuss, 2001). In addition to reducing hypovirulence, traits of phenotype can also be altered in hypovirus-infected *C. parasitica* strains, such as suppressed sporulation, decreased pigmentation, and altered gene expression patterns (Nuss, 2005; Eusebio-Cope et al., 2015). In this report, we observed that both hypovirus CHV1-EP713 infection and treatment with the autophagy-inducing drug rapamycin in the wild-type strain EP155 could cause proliferation of autophagic-like vesicles, and expression of autophagy-related genes was up-regulated following infection by hypovirus CHV1-EP713. Disruption of *cpatg8*, a gene encoding a homolog of *ATG8* in *S. cerevisiae*, caused a profound reduction in fungal virulence, conidiation, and accumulation of the virus.

## Materials and Methods

### Fungal Strains and Growth Conditions

*C. parasitica* wild-type strain EP155 (ATCC38755), its isogenic strain EP155/CHV1-EP713 harboring hypovirus CHV1-EP713 by transfection, and strain DK80, a *ku80*-deletion mutant of EP155 and highly efficient in gene homologous replacement (Lan et al., 2008; Choi et al., 2012), as well as *cpatg8* deletion strain Δ*cpatg8* were all maintained on potato dextrose agar (PDA) medium (Difco, Detroit, MI) at 24–26°C with a 12 h/12 h light/dark cycle (1,300–1,600 lx), as described previously (Chen et al., 2011). EP complete medium was employed for cultures used for DNA and dsRNA isolation at room temperature with shaking at 200 rpm for 3 days. The transformation of *C. parasitica* was done as described (Chen et al., 2011). Hygromycin (40 μg/ml) or G418 (25 μg/ml) was supplemented into the growth medium for selection of transformants.

### Gene Manipulation

Gene cloning, PCR, and Southern analysis were performed according to Sambrook and Russell (2001). Primers used were synthesized by Sangon Biotech Co., Ltd. (Shanghai, China) and listed in Table 1.

**Table 1:** List of primers used.

**Primer name**	**Sequence 5^**′**^-3^**′**^**	**Name of gene**
Hyg-F	CTGAAATAAAGGGAGGAAGGG	*hph*
Hyg-R	AGGACACACATTCATCGTAGG	
*cpatg8*-all-F	CGTGGGGTGACTTTGAGAGTGA	*cpatg8*
*cpatg8*-all-R	CTTGCCTACGAGGTCACTGGTCA	
*cpatg8*-LF	TACTTCTTCTGCCTGCCTTTGGG	*cpatg8*
*cpatg8*-LR	ATATCATCTTCTGTCGACCTGCAGGCCGGTGGTCGGTGAAAGTAGGGT	
*cpatg8*-RF	TCTTTCTAGAGGATCCCCGGGTACCGATAGCGGGTGTTCGTTCTTCTGC	*cpatg8*
*cpatg8*-RR	GTCTCATGTCGCCGGGTACATG	
Δ*cpatg8*-com-F	CGAGAATTCCACTTGGGTACTGCTGGC	*cpatg8*
Δ*cpatg8*-com-R	TATGCGGCCGCGATTGACTCAAAGTCTC	
18S-F	TCTCGAATCGCATGGCCT	18S rRNA
18S-R	TTACCCGTTGTAACCACGGC	
*cpatg1*-F	TCCACAACCTGTGCCATCCACTTCA	*cpatg1*
*cpatg1*-R	TTGTCGACCACGACATAGTCACGCT	
*cpatg3*-F	GGCCTCGGTGCACCCTTGCA	*cpatg3*
*cpatg3*-R	ATGAACTTGAGGAACACCAC	
*cpatg4*-F	CGCTCGACAAGAACGTGAGA	*cpatg4*
*cpatg4*-R	GTATCAGTGTTGGATGGAATG	
*cpatg7*-F	GCGTCGACAACAGGGAATA	*cpatg7*
*cpatg7*-R	AGAGACGAAGCGGTCCTCCTC	
*cpatg8*-F	ATCCAAGTTCAAGGATGAGC	*cpatg8*
*cpatg8*-R	AGATGGCCTTGTCGGGGGAC	
*cpatg18*-F	CTCGTCACCGCGTGCGAATCG	*cpatg18*
*cpatg18*-R	ACCGCTCTGTCTCCGATTTG	
*cpatg33*-F	ACAACAGTCCCGCAAGGACCGC	*cpatg33*
*cpatg33*-R	TGCTTCTTGAGGAAGTCCTCG	
p29-F	ATAGCGGCCGCATGGCTCAATTAAGAAAACCC	p29
p29-R	ATAGTTAACTTAGCCAATCCGGGCAAGGGGATC	

### Construction and Complementation of *cpatg8* Null Mutants

*cpatg8* null mutants were constructed by homologous recombination. Briefly, a fragment containing a hygromycin-resistant gene *hph* in place of the *cpatg8* coding region that was flanked with *cpatg8* sequences was generated by PCR. This fragment was introduced into DK80 spheroplasts *via* the PEG-mediated transformation protocol. Putative *cpatg8* disruptants were screened by PCR, selected for nuclear homogeneity by single-spore isolation, and further verified by Southern blot analysis. Confirmed transformants were designated as Δ*cpatg8* strains. A 2.55 kb genomic fragment with *EcoR*I and *Not*I containing the complete *cpatg8* transcript region (0.69 kb), promoter region (1.20 kb), and terminator region (0.66 kb) was amplified by PCR and then inserted into the transformation vector pCPXG418 to generate the construct pCPXG418-*cpatg8*. Complemented strains were constructed by transforming Δ*cpatg8* spheroplasts with pCPXG418-*cpatg8* and the complemented transformants were validated by PCR and Southern blot.

### Construction of GFP-Labeled CpATG8 and RFP-Labeled p29 Strains

GFP-CpATG8 fusion plasmid (pCPXG418-GFP-*cpatg8*) was constructed as described (Shi et al., 2014). CpATG8 encoding region sequence was amplified and cloned into the pCPXG418-GFP to construct pCPXG418-GFP-*cpatg8*. Likewise, the virus-encoded protein p29 encoding region sequence was amplified by PCR with primer pair *p29*-F/R (Table 1) and cloned into the pCPXHY2-RFP to generate the recombinant plasmids pCPXHY2-RFP-p29. Subsequently, these two recombinant plasmids were transformed into the protoplasts of the wild-type strain EP155, respectively. The expression of GFP and RFP was observed using an Olympus BX51 fluorescence microscope.

### Electron Microscopy

For scanning electron microscopy, fungal samples (2 × 4 mm) were prepared after 5 days of cultivation on PDA. Samples were fixed in 2% (v/v) glutaraldehyde in 0.2 M phosphate buffer (pH 6.8) at 4°C for 4–6 h and then washed with the same buffer for 2 h. The samples were dehydrated in a graded acetone series (30, 50, 70, 80, 90, and 100%) with each grade kept for 30 min and three times in 100% acetone. Finally, the fully dehydrated samples were dried in a Critical Point Dryer (HCP-2, Hitachi), mounted on stubs, and then coated with gold about 200 nm in thickness in a Sputter Coater (S-3400N, Hitachi). The coated specimens were observed with a SEM HV (S-3400N, Hitachi) at 10 kV.

For transmission electron microscopy, hyphae cultivated on PDA medium for 7 days were scraped with a clean scalpel and washed three times with sterilized distilled water, fixed with 2.5% glutaraldehyde in 0.1 M phosphate buffer (pH 7.2) at 4°C overnight, rinsed three times with phosphate buffer (50 mM, pH6.8), and post-fixed overnight in 1% osmium tetroxide in 0.1 M cacodylate buffer (pH 7.0) at 4°C for 2 h. After rinsing with phosphate buffer, the samples were dehydrated in a gradient ethanol series and embedded in Epon 812 resin. The ultrathin sections were stained in 2% uranium acetate followed by lead citrate and visualized under a transmission electron microscope (Hitachi, H-7650) operating at 80 kV.

### Quantification of Gene Transcripts and Viral dsRNA

The relative accumulation of gene transcripts in the strain DK80 and Δ*cpatg8* was measured using quantitative real-time RT-PCR as previously described (Shi et al., 2014). Total cDNA was synthesized using an amount of 4 μg of RNA with appropriate gene-specific primers (Table 1). The real-time PCR was performed in a LightCycler 480 (Roche Applied Science) and normalized against that of 18S rRNA. The viral dsRNA accumulation level was examined using the method described previously (Lin et al., 2007). RNA samples stained with ethidium bromide were scanned using a Typhoon 9410 phosphorimager (GE Healthcare Life Sciences). To quantify the relative amount of large and medium dsRNA, the scanned gel image analysis was performed by ImageQuant TL-1D gel analysis software. 18S rRNA was used as the normalization reference.

### Virulence Assays

Virulence was tested on dormant stems of Chinese chestnut (*Castanea mollissima*) with five replicates per fungal strain as previously described (Yao et al., 2013). The inoculated stems were kept at room temperature in a plastic bag to maintain moisture for 4 weeks. After incubation for 4 weeks, canker sizes were measured and the results were subjected to statistical analysis using the PROCGLM procedure (SAS, version 8.0). The type I error rate was set at *P* < 0.05.

## Results

### Hypovirus Infection Promotes Autophagy in *C. parasitica*

**Figure 1:**
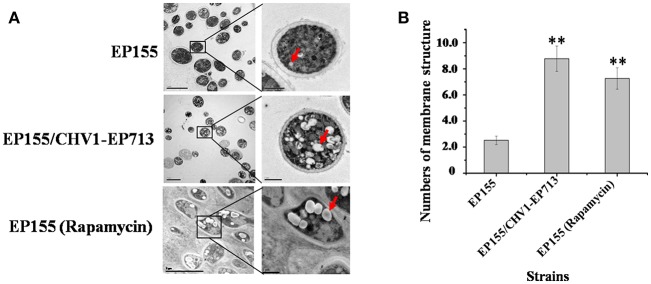
Hypovirus infection and rapamycin treatment resulted in the accumulation of autophagosome-like vesicle. **(A)** Transmission electron micrographs of the hyphae. The hypha morphology of EP155 and EP155/CHV1-EP713 did not show apparent distinction. However, the intracellular structure of EP155/CHV1-EP713 showed much more membrane structures. Arrow indicates autophagosome-like vesicle. **(B)** The quantification of the average number of autophagosome-like vesicles per cell and a minimum of 20 cells were counted. Rapamycin-treated EM graphs (14 days on rapamycin-supplemented PDA plate). ^**^ indicates *P* < 0.001, determined by Student's *t*-test.

Previous studies showed that CHV1-EP713 and virus-encoded protein p29 were presented in vesicles (Dodds, 1980; Jacob-Wilk et al., 2006; Wang et al., 2013). We used an electron microscope to compare the subcellullar structure of the wild-type strain EP155 and virus-infected strain EP155/CHV1-EP713 and found that the number of vesicles was increased in the cytoplasm by more than 3-fold following virus infection (Figure 1).

**Figure 2:**
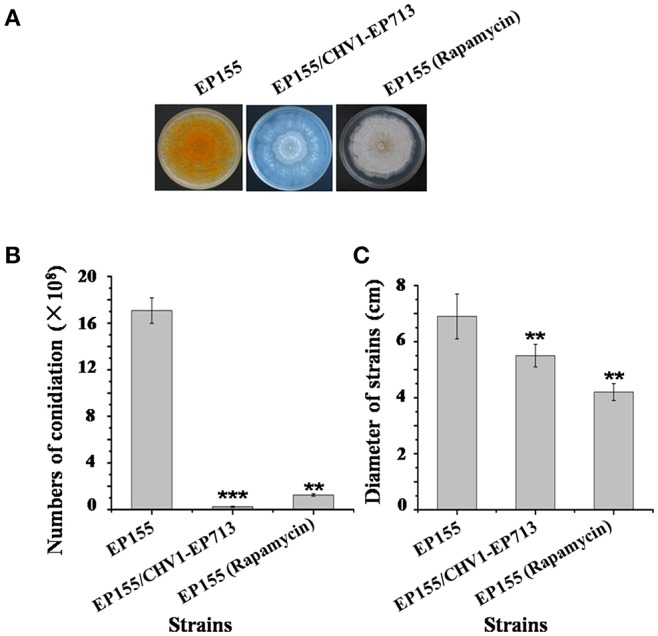
Sporulation level and growth rates of hypovirus infection and rapamycin treatment on *C. parasitica*. **(A)** Phenotypes on PDA. Photo was taken at day 7. **(B)** Sporulation level. Spores were counted on day 14. **(C)** Growth rates of EP155, EP155/CHV1-EP713, and EP155 (rapamycin treatment, 60 nM) Growth rate was measured from cultures grown on PDA for 7 days. Values are means ± S.E.M of three independent experiments. ^**^ indicates *P* < 0.01 and ^***^ indicates *P* < 0.001, determined by Student's *t*-test.

**Figure 3:**
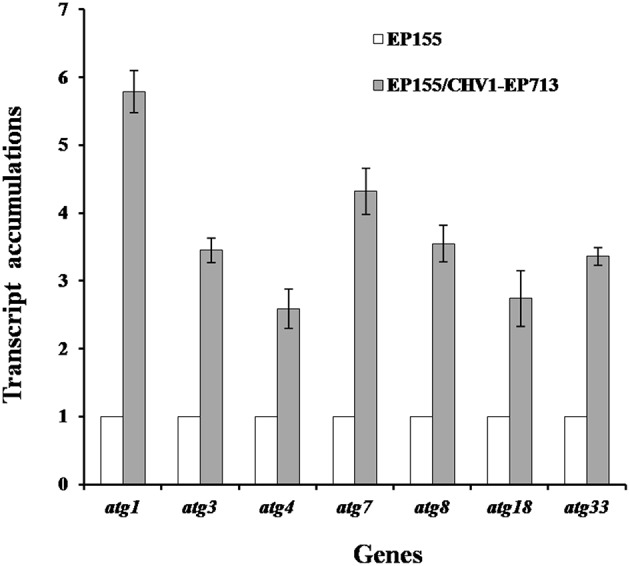
Transcript accumulation levels of autophagy-related genes. The transcript accumulation level for each of the target genes in EP155 was set at 1.0, and the corresponding levels in EP155/CHV1-EP713 were expressed as a percentage of that of EP155. Values were calculated from three biological repeats. Bars indicate mean deviations.

Rapamycin can induce autophagy (Klionsky et al., 2016). EP155 treated with rapamycin resulted in a similar increase in vesicle numbers (Figure 1). Moreover, rapamycin-induced EP155 exhibited the phenotype similar to that of EP155/CHV-1EP713, including decreased sporulation, growth, and pigment production (Figure 2). To understand whether the autophagy pathway could be induced upon CHV1-EP713 infection, qRT-PCR was performed to compare transcripts of autophagy-related genes. Results showed that expression of *cpatg1, cpatg3, cpatg4, cpatg7, cpatg8, cpatg18*, and *cpatg33* was significantly up-regulated (*P* < 0.05) by 5.79-, 3.45-, 2.59-, 4.32-, 3.55-, 2.74-, and 3.36-fold, respectively (Figure 3). Thus, it was concluded that virus infection stimulated autophagy in *C. parasitica*.

### *cpatg8* Is Essential for Autophagy in *C. parasitica*

The putative *atg8* homolog was inspected against the *C. parasitica* genome database (http://genome.jgi-psf.org/cgi-bin/dispGeneModel?db=Crypa2&id=102797). The coding region of the *cpatg8* gene is composed of three exons with 124 amino acid residues and two introns of 314 bp. The deduced CpATG8 protein showed a high level of homology (94–97% amino acid identity) compared to those of *Magnaporthe oryzae, Neurospora crassa, Pichia pastoris*, and *Aspergillus nidulans*, with *Chaetomium thermophilum* and *M. oryzae* being the highest at 97% and *A. nidulans* being the lowest at 94% (Figure S1).

**Figure 4:**
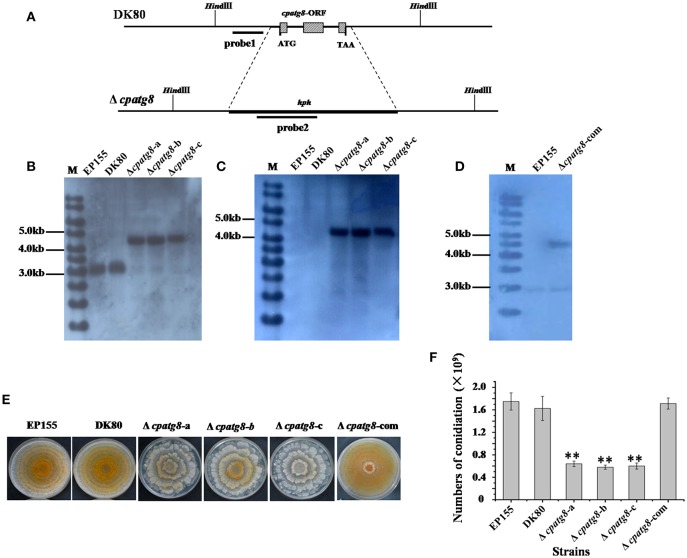
Phenotypes and Southern analysis of *cpatg8* knockout mutants. **(A)** Diagram of *cpatg8* gene replacement strategy; probe fragment on the left arm was used in the Southern blot analysis to distinguish the fragment size of the wild-type strain and *cpatg8* null mutants; Southern analysis of the *cpatg8* null mutants **(B,C)** and complementary strain **(D)**. Fungal total DNAs were digested with *Hind* III and separated on a 0.8% agarose gel by electrophoresis, and blotted using probe 1 **(B)** and probe 2 **(C)**, respectively. Fragment sizes are indicated in the figure margins. **(E)** Mutant colony morphologies on PDA plates. Fungal strains were cultured on the laboratory bench top condition at 24°C, and the photograph was taken on day 14 postinoculation. **(F)** Sporulation level of *cpatg8* knockout mutants. Spores were counted on day 14. Values are means ± S.E.M of three independent experiments. ^**^ indicates *P* < 0.01, determined by Student's *t*-test.

To investigate the functions of the *cpatg8* gene, *cpatg8* disruption mutants were constructed *via* homologous recombination with a hygromycin-B-resistant cassette (Figure 4A). Three randomly selected single-spore-derived transformants were screened by PCR and further confirmed by Southern blot analysis (Figures 4B–D). The conidiation level of the Δ*cpatg8* was drastically reduced and aerial hyphae were significantly less than those of the parent strain DK80 and wild-type strain EP155 (Figures 4E,F). As shown in Figure 4E, the day–night growth patterns of Δ*cpatg8* were different from its parental or the wild-type strain. The abnormal phenotype of the mutants could be fully restored by reintroducing a wild-type copy of *cpatg8* (Figure 4E), suggesting that *cpatg8* is solely responsible for the altered phenotype.

**Figure 5:**
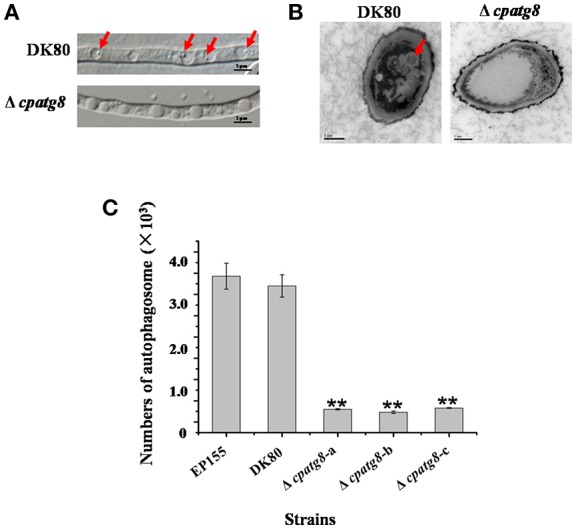
Electron micrographs of the hyphae of strain DK80 and Δ*cpatg8* mutant. **(A)** Autophagy in the aerial hyphae of *C. parasitica*. Autophagic bodies in the vacuoles of the aerial hyphae of the strain DK80 and Δ*cpatg8* mutant grown on plates of PDA were examined using differential interference microscopy. **(B)** Autophagy was blocked in Δ*cpatg8* mutant. Vacuoles in the hyphae of the parental strain DK80 and Δ*cpatg8* mutant were observed using an electron microscope after being cultured in EP liquid media in the presence of 2 mM PMSF for 4 h (bar, 0.5 μm). **(C)** The quantification of the number of autophagic bodies. Arrow indicates autophagic body (bar, 5 μm). Values are means ± S.E.M of three independent experiments. ^**^ indicates *P* < 0.01, determined by Student's *t*-test.

Using differential interference microscopy and transmission electron microscopy, we examined changes in the process of autophagy stabilized by addition of phenylmethylsulfonylfluoride (PMSF) (Klionsky et al., 2016) in the Δ*cpatg8* mutants and DK80 by differential interference microscopy. Only 8.08 ± 2.35% of the vacuoles had autophagic bodies in the Δ*cpatg8* mutants, whereas it was 79.13 ± 8.21% in DK80, when cultured in EP liquid medium in the presence of 2 mM PMSF for 4 h (Figures 5A,C). Autophagic bodies in vacuoles of the strain DK80 were seen by transmission electron microscopy, but not in the *cpatg8* mutant (Figure 5B), suggesting that *cpatg8* is a gene essential for autophagy in *C. parasitica*.

### Deletion of *cpatg8* Attenuates *C. parasitica* Virulence and Reduces Accumulation of the Hypovirus RNA

**Figure 6:**
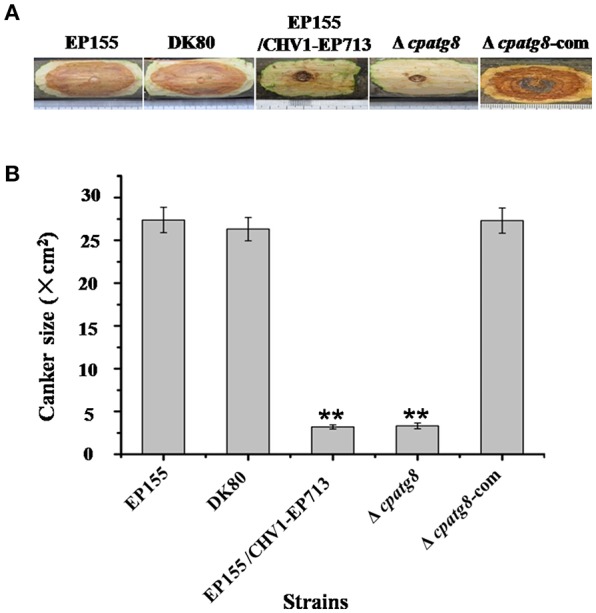
Virulence assay on chestnut stems. **(A)** Cankers induced by the tested strains. The wild-type (EP155), starting (DK80), and hypovirus-infected (DK80/CHV1-EP713) strains and *cpatg8*-deleted (Δ*cpatg8*) and *cpatg8*-complemented (Δ*cpatg8*-com) mutants were inoculated onto Chinese chestnut (*C. mollissima* Blume) stems. The inoculated stems were kept at 24°C and cankers were measured and photographed on day 28 postinoculation. **(B)** Canker size measurements of the tested strains. The assays were with five duplicates for each strain. Values are means ± SEM of three independent experiments. ^**^ indicates *P* < 0.01, determined by Student's *t*-test.

EP155 and parental strain DK80 were highly virulent and incited large cankers on chestnut stems, whereas Δ*cpatg8* caused very small cankers, similar to those of EP155/CHV1-EP713. Virulence of the Δ*cpatg8* mutant could be fully restored following reintroduction of the wild-type cpatg8 gene (Figure 6).

**Figure 7:**
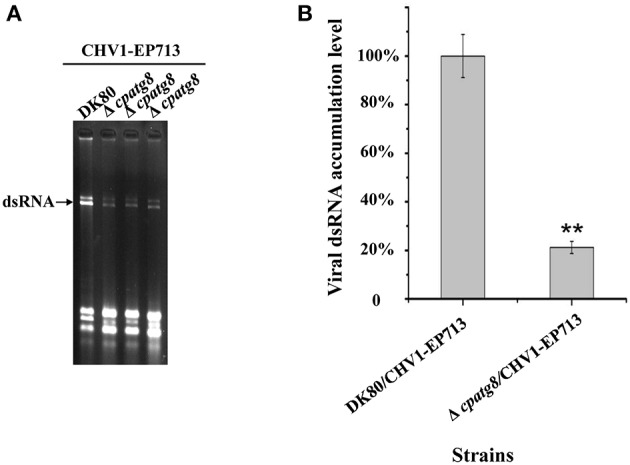
Quantification of viral dsRNA in Δ*cpatg8* mutant transfectants. All transfectants were in the DK80 genetic background. **(A)** Agarose gel electrophoretic analysis of viral dsRNA. Ten micrograms of total RNA from virus-infected hosts was loaded into each lane in a 1.0% agarose gel. Lane 1, DK80/CHV1-EP713; lane 2 to lane 4, Δ*cpatg8* mutant transfectants with CHV1-EP713. The arrow indicates the position of viral dsRNA. **(B)** Semi-quantification of viral dsRNA from different transfectants. The viral dsRNA level of EP155/CHV1-EP713 was set at 100%, and levels of Δ*cpatg8* mutant transfectants were expressed as percentages of that of CHV1-EP713. Values are means ± S.E.M of three independent experiments. ^**^ indicates *P* < 0.01, determined by Student's *t*-test.

*atg8* is known to be a key gene of autophagy that functions at different stages of the autophagy pathway (Klionsky et al., 2016). To conclusively establish whether autophagy was required for replication of CHV1-EP713, Δ*cpatg8* was paired with hypovirus-infected strain EP155/CHV1-EP713. While the converted Δ*cpatg8* colonies showed viral-infected phenotypes of reduced conidiation and loss of pigmentation, the accumulation level of the viral dsRNA was significantly reduced (Figure 7), demonstrating that *cpatg8* plays an important role in CHV1-EP713 replication.

### Viral Protein p29 Co-localizes With CpATG8

**Figure 8:**
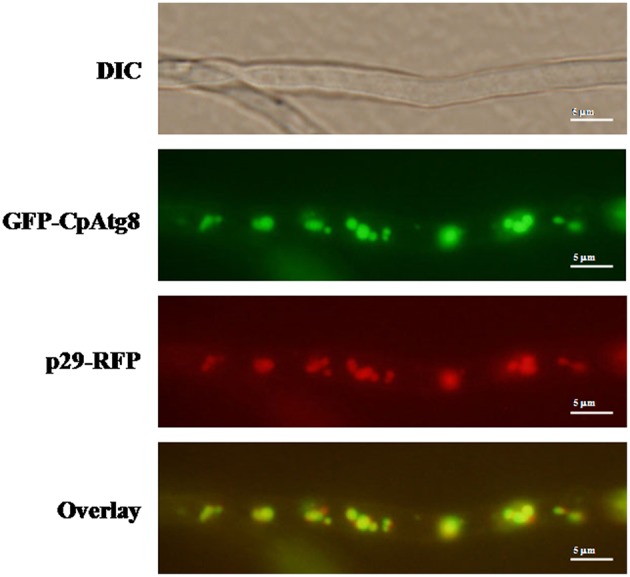
Localization of p29-RFP and GFP-CpATG8 under normal growth conditions. The strain expressing p29-RFP and GFP-CpATG8 was grown on PDA medium. After 7 days, hyphae were observed by DIC and fluorescence microscopy. Scale bars, 5 μm.

ATG8 has been used as a marker for autophagy-related structures in a wide range of eukaryotes (Pollack et al., 2009; Voigt and Pöggeler, 2013a; Yin et al., 2016). By co-expression of GFP-CpATG8 and viral p29-RFP in EP155, it was observed that these two proteins co-localized in the vesicles of the cell (Figure 8), likely in the autophagosomes.

## Discussion

Autophagy is an evolutionarily conserved biological process found in eukaryotic cells involved in recycling processes. Autophagy can be measured by using fluorescent marker-tagged Atg8 and be inhibited by deletion of autophagy-related genes (Veneault-Fourrey et al., 2006; Duan et al., 2013; Sumita et al., 2017; Ren et al., 2018). Consistently, loss of *cpatg8* leads to the inhibited autophagy in *C. parasitica* (Figure 4), suggesting that *cpatg8* is essential for autophagy in this fungus.

Disruption of *atg8* has been reported to result in reduced conidiation, impaired aerial mycelial growth, and attenuated virulence in several pathogenic fungi. In the rice blast fungus *M. oryzae*, it was shown that autophagy was necessary for the blast disease (Kershaw and Talbot, 2009). Disruption of *MgATG8* resulted in autophagy-arrested conidial cell death and loss of virulence (Veneault-Fourrey et al., 2006; Liu et al., 2010). In the corn smut fungus *Ustilago maydis*, autophagy was required for pathogenicity (Nadal and Gold, 2010). In *Botrytis cinerea, BcATG8* is essential for autophagy to regulate fungal development and pathogenesis, and deletion of *BcATG8* blocked autophagy and significantly impaired aerial hyphal growth, reproductive development, and virulence (Ren et al., 2018). In this regard, our current study was in accordance with the previous findings that ATG8 is required for conidiation and virulence.

Induction of autophagy and exploitation of components of autophagy pathway in favor of viral replication and spread have been reported for many RNA and DNA viruses. For example, HIV blocks the formation of mature autolysosomes in macrophages and exploits the autophagic component during early stages in replication (Kyei et al., 2009). Poliovirus uses autophagy components for genome replication, while dengue and Zika viruses use autophagy components for post-replication processes (Abernathy et al., 2019). In our study, accumulation of autophagosome-like vesicle was found in both CHV1-EP713-infected and rapamycin-treated strains (Figure 1), and genes involved in autophagy were up-regulated in hypovirus-infected strain (Figure 3), suggesting that hypovirus may induce and exploit autophagy for its genome replication. Reduced accumulation of hypoviral dsRNA in cpatg8 null mutant (Figure 7) further supports this assumption. As a matter of fact, it has been reported that hypovirus infection induces proliferation of the vesicle in *C. parasitica* (Dodds, 1980; Wang et al., 2013) and hypovirus dsRNA and viral encoded p29 had been found to co-fractionate with a trans-Golgi network-derived membrane (Jacob-Wilk et al., 2006), implying that the hypovirus may be trafficked by the vesicles.

The hypovirus-encoded p29 is a multifunctional protein involved in virus replication, suppression of host RNA interference system, post-transcription modification, sporulation, host symptom development, and virulence (Choi et al., 1991; Suzuki et al., 2003; Andika et al., 2019). Our studies also showed hypovirus CHV1-EP713 protein p29-RFP co-localized with GFP-CpATG8. Whether p29 is involved in the process of autophagosome fusion with lysosomes remains to be determined.

## Author Contributions

LS carried out the experiment and helped to drafted the manuscript. JW participated in the culture of fungal strains, data analysis, and helped to draft the manuscript. RQ and FY participated in the culture of fungal strains and data analysis. JS designed and supervised the experiment and drafted the manuscript. BC designed and supervised the experiment and revised the manuscript.

### Conflict of Interest Statement

The authors declare that the research was conducted in the absence of any commercial or financial relationships that could be construed as a potential conflict of interest.
