# Supplementary material for: CpATG8, a Homolog of Yeast Autophagy Protein ATG8, Is Required for Pathogenesis and Hypovirus Accumulation in the Chest Blight Fungus
Source: Front Cell Infect Microbiol. 2019 Jul 10;9:222. doi: 10.3389/fcimb.2019.00222 (PMC6635641; doi:10.3389/fcimb.2019.00222)

**Supplementary Material**

**Figure S1.** Autophagy in the aerial hyphae of *C. parasitica*. Autophagic bodies in the vacuoles of the aerial hyphae of the strain DK80 and Δ*cpatg8* mutant grown on plates of PDA were examined using differential interference microscopy. Scale bars, 5 μm.
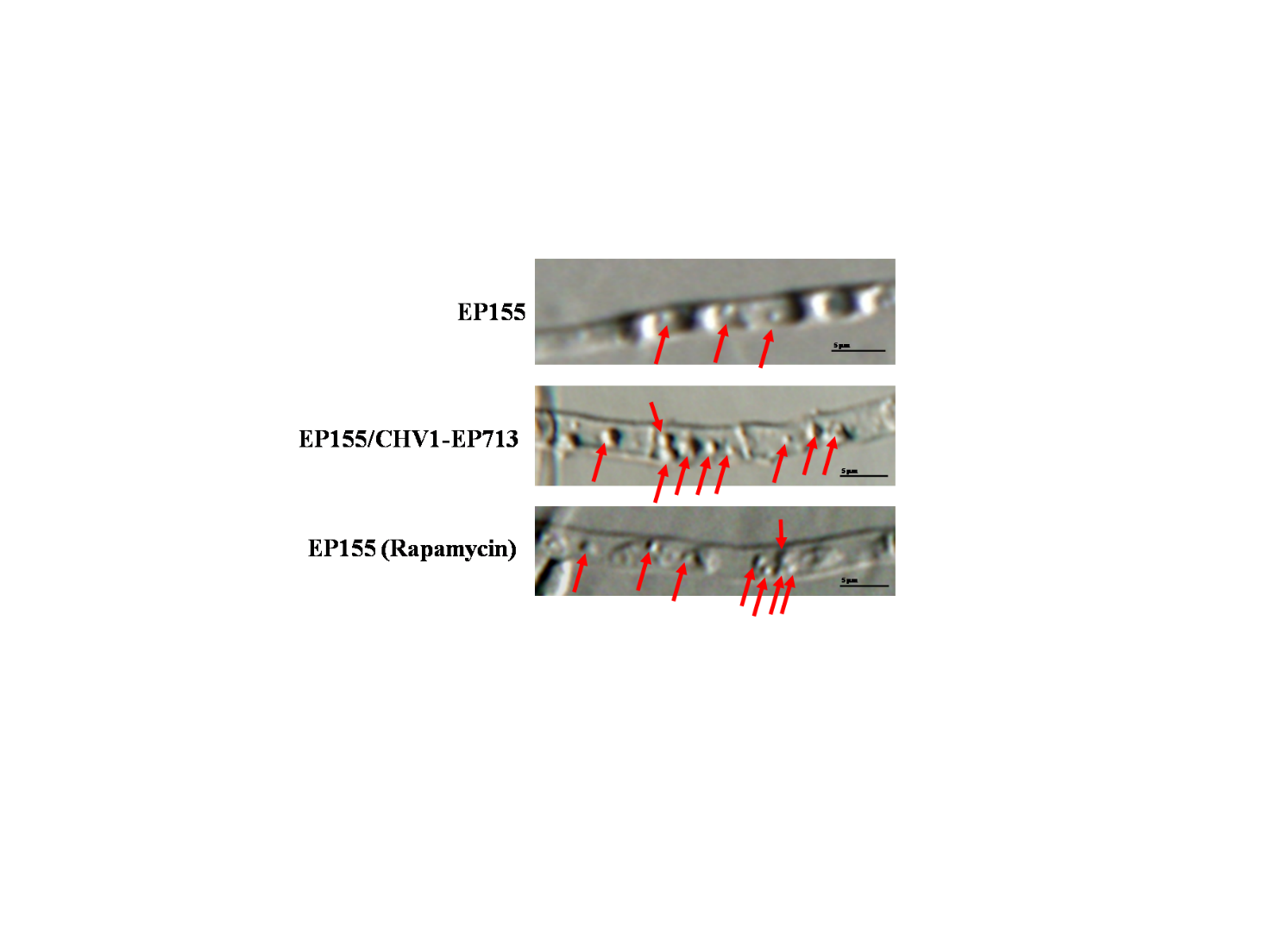

Supplement: Supplementary file 1 [file Data_Sheet_1.docx]
